# Genetic risk for treatment resistant schizophrenia and corresponding variation in dopamine synthesis capacity and D_2/3_ receptor availability in healthy individuals

**DOI:** 10.1038/s41380-024-02873-2

**Published:** 2024-12-27

**Authors:** Daniel Paul Eisenberg, Rachael Keir Blackman, Maria G. Tietcheu, Philip D. Kohn, Jasmin S. Bettina, Bhaskar Kolachana, Michael D. Gregory, Karen F. Berman

**Affiliations:** 1 From the Clinical & Translational Neuroscience Branch, Intramural Research Program, National Institute of Mental Health, NIH, DHHS, Bethesda, MD 20892 USA; 2 Human Brain Collection Core, Intramural Research Program, National Institute of Mental Health, NIH, DHHS, Bethesda, MD 20892 USA

**Keywords:** Schizophrenia, Genetics

## Abstract

Dysfunction of dopamine systems has long been considered a hallmark of schizophrenia, and nearly all current first-line medication treatments block dopamine D_2_ receptors. However, approximately a quarter of patients will not adequately respond to these agents and are considered treatment-resistant. Whereas abnormally high striatal presynaptic dopamine synthesis capacity has been observed in people with schizophrenia, studies of treatment-resistant patients have not shown this pattern and have even found the opposite – i.e., *reductions* in striatal presynaptic dopamine synthesis capacity. Whether such reductions in fact represent clinical epiphenomena such as medication or other treatment effects or whether they rather represent neurobiological differences related to etiology has been unclear. To understand the dopaminergic implications of genetic liability for treatment-resistant schizophrenia without the confound of clinical epiphenomena, we studied a cohort of healthy individuals without neuropsychiatric illness using [^18^F]-FDOPA positron emission tomography (PET) and found that striatal presynaptic dopamine synthesis capacity showed an expected direct association with cumulative genetic risk burden for general schizophrenia but an *inverse* association with specific polygenic risk for treatment-resistant schizophrenia. Subsequent evaluation of D_2/3_ dopamine receptor availability in an overlapping cohort using [^18^F]-fallypride PET did not identify any effects of genetic risk in the striatum but found an association with treatment-resistant schizophrenia polygenic risk in the thalamus. Overall, these results align with prior PET studies in patients and implicate, at least with respect to the dopamine system, fundamentally distinct molecular mechanisms in the unique genetic liability for treatment-resistant schizophrenia.

## Introduction

Antipsychotic medication treatment is a critical component of effective care in schizophrenia and can provide relief for the many symptoms of this disorder; however, nearly a quarter of individuals do not show significant improvement with first-line agents and are considered treatment-resistant [1]. Aside from clozapine, a unique medication with superior efficacy over other available antipsychotic drugs but requiring careful monitoring for serious side effects [2], evidence-supported treatment options for individuals with treatment resistance are limited. Critically, an inadequate understanding of the core neurogenetics and neurobiology of treatment-resistant schizophrenia (TRS) continues to hamper the design of novel therapeutics.

Dysfunction of the dopamine systems has long been considered a hallmark of schizophrenia spectrum illness [3] and, despite therapeutic roles for additional neurotransmitters, is a target of all current first-line medication treatments for this condition [4]. The observations that high-dose dopamine agonists can induce or potentiate psychotic symptoms and that dopamine D_2_ receptor blockade has antipsychotic effects are foundational elements of the modern understanding of schizophrenia neurobiology [5, 6]. Molecular imaging experiments have found evidence for excessive presynaptic dopaminergic drive in psychosis, identifying, on group-average, abnormally high amphetamine-induced dopamine release [7, 8] and striatal presynaptic dopamine synthesis capacity [3] in patients. The latter observation has also been seen in first-degree relatives [9] and individuals at-risk for schizophrenia [10], suggesting that elevated striatal presynaptic dopamine synthesis capacity, as is measured by [^18^F]-FDOPA positron emission tomography (PET), could be an important genetically-mediated intermediate phenotype in schizophrenia. However, across patients, this phenotype shows substantial heterogeneity, and many individuals with schizophrenia spectrum illness do not show elevated striatal presynaptic dopamine synthesis capacity, with clinical factors underlying some of this variability [11]. Importantly, treatment non-response has been associated with relatively *lower* presynaptic dopamine synthesis capacity [12], and in one study, individuals with TRS receiving clozapine treatment showed abnormally *low* presynaptic dopamine synthesis capacity compared to controls [13]. If striatal dopaminergic tone tends to be greater in schizophrenia generally but is instead lower in TRS, it may be that major neurobiological differences underly TRS etiology or pathophysiology. Alternatively, medication (e.g., clozapine) effects or other clinical epiphenomena unique to TRS cohorts could be significant drivers of these opposing phenotypes.

Recent meta-analytic work leveraging large-scale consortia genetic data from case-control studies of non-TRS (i.e., unselected for treatment responsiveness) and clozapine-treated TRS has been able to identify differences between TRS and non-TRS risk allele effect sizes across the genome, resulting in a statistical assessment of genetic risk variation specifically associated with TRS [14]. The biological implications of this variation remain undetermined but are important to identify. An understanding of how genetic vulnerability to TRS manifests in the living brain may ultimately yield insights into illness nosology, novel treatment targets, and cohort stratification for treatment trials.

Here, we investigate the possible sequelae of cumulative schizophrenia risk genetics without the confounds of TRS-associated clinical epiphenomena such as medication exposure or frequent hospitalization by examining a large cohort of healthy individuals who provided genetic material for analysis and underwent dopaminergic PET neuroimaging. We hypothesized that if general schizophrenia (i.e., non-TRS) risk neurobiology and TRS risk neurobiology predispose to divergent dopamine system properties as suggested by prior clinical studies, then greater non-TRS polygenic risk burden would predict greater striatal presynaptic dopamine synthesis in this cohort, whereas greater TRS polygenic risk burden would predict less. Furthermore, because D_2_ dopamine receptors are the primary target of mainstay antipsychotic treatments and because schizophrenia has been associated with both diminished thalamic [15] and elevated striatal [3] D_2/3_ receptor availability in meta-analytic studies, we secondarily tested the hypothesis that TRS polygenic risk burden might also be associated with differences in D_2/3_ dopamine receptor availability.

## Methods

### Participants

Two-hundred-and-two healthy adults (18–59 years of age) without psychiatric illness were studied at the U.S. National Institutes of Health (NIH) Clinical Center. Of these, 187 participated in [^18^F]-FDOPA neuroimaging to measure presynaptic dopamine synthesis capacity (98 [52.4%] female, mean age 35.7 ± 11.1 years), and 104 participated in [^18^F]-fallypride neuroimaging to measure dopamine D_2/3_ receptor availability (47 [45.2%] female, mean age 38.1 ± 11.1 years). All participants were of European decent and were free of psychiatric diagnosis, neurological condition, substance use disorder or other confounding medical conditions as determined by clinical evaluations, including history and physical examination, psychiatric diagnostic interview [16], laboratory testing, and clinical magnetic resonance neuroimaging.

### Genetics

Genotyping, quality control, and imputation procedures were conducted as previously reported [17]. Participants provided peripheral venous blood samples, and DNA from mononuclear cells was extracted for genotyping, which was conducted with Illumina BeadChips (510K–2.5 M SNP chips). Quality control was performed in standard fashion with all samples demonstrating SNP missingness <0.05 (before sample removal); subject missingness <0.02; autosomal heterozygosity deviation (|Fhet|<+/−3.5 standard deviations); SNP missingness <0.02 (after sample removal), SNP Hardy-Weinberg equilibrium (HWE) *p* > 10^−6^; minor allele frequency (MAF) > 0.01; and identity by descendent threshold of 0.185. Data were phased using Shapeit software (https://mathgen.stats.ox.ac.uk/genetics_software/shapeit/shapeit.html), and imputation used IMPUTE2 (https://mathgen.stats.ox.ac.uk/impute/impute_v2.html) with default parameters and a chunk size of 250 Kb. The 1000 Genomes Phase 3 data were used as a reference panel for the Illumina HumanOmni2.5-v1.2 chip, and the resultant imputation served as the reference panel for the remaining smaller chips. The genomic data were subjected to principal component analysis using PLINK software (https://www.cog-genomics.org/plink/1.9) with the first three components retained for use as population stratification covariates.

Polygenic scores both for unselected schizophrenia (non-TRS) risk and for differential TRS risk relied upon summary statistics derived from two recent, large-scale genetic association studies: the Psychiatric Genomics Consortium “Wave 3” schizophrenia genome-wide association study meta-analysis [18] and the treatment-resistance interaction meta-analysis of Pardiñas and colleagues, which evaluated cohorts of individuals who had been prescribed clozapine and had evidence for failure of at least 2 other antipsychotic medications [14]. PRSice-2 software (https://choishingwan.github.io/PRSice/) was employed to calculate polygenic risk scores both for non-TRS and for TRS at multiple p-value thresholds (ranging from 5 × 10^−8^ to 1.0), which were then reduced using principal components analysis with R software (https://www.r-project.org/), as previously described [19]. Resultant primary (first component) summary measures of risk proclivity for non-TRS and TRS were then forwarded for further analysis.

### Neuroimaging

#### Acquisition procedures

All scans were performed after a four-hour minimum abstinence period from caffeine and nicotine. Additionally, for [^18^F]-FDOPA scans, a six-hour minimum fast (to prevent amino acid-mediated competition for tracer transport across the blood brain barrier) was required, and participants were administered carbidopa 200 mg by mouth approximately one hour prior to [^18^F]-FDOPA injection (to prevent peripheral tracer decarboxylation). For [^18^F]-FDOPA scans, a General Electric Advance PET camera was used in 3D mode. An individualized thermoplastic mask was contoured to each participant’s head to limit movement. After head position was established in the camera bore, a ^68^Ge transmission scan for attenuation correction was performed. [^18^F]-FDOPA emission scans immediately followed bolus tracer injection (target dosing of up to 16 mCi; mean dose 15.3 ± 2 mCi; mean specific activity 1180 ± 393 mCi/mmol) and were collected in dynamically binned frames over approximately 90 min.

All [^18^F]-fallypride scans were carried out on a Siemens ECAT HRRT camera outfitted with a Northern Digital Polaris Vicra optical measurement system. Prior to scanning, participants donned a cap with attached spherical reflectors, which permitted head motion tracking. After head position was established in the camera bore, a ^137^Cs transmission scan for attenuation correction was performed. [^18^F]-fallypride emission scans began immediately following bolus tracer injection (target dosing of up to 5 mCi; mean dose 5.1 ± 0.2 mCi; mean specific activity 2603 ± 1249 mCi/μmol) and were collected over a period of approximately four hours, which included two brief planned breaks for the participant out of the scanner. Re-positioning and transmission scanning preceded emission scanning upon the participant’s return to the gantry following each break.

In separate imaging sessions, participants underwent T1-weighted structural magnetic resonance imaging (MRI) at 3T for coregistration and spatial warping purposes.

#### Data processing procedures

For [^18^F]-FDOPA scans, reconstruction with filtered back-projection with registered attenuation correction that adjusted for frame-wise head motion was performed. For [^18^F]-fallypride scans, reconstruction with ordered subset expectation maximization was conducted, with head tracking data used to correct for motion.

Each participant’s T1-weighted structural MRI volumes were averaged, intensity normalized [20] and segmented using Freesurfer (https://surfer.nmr.mgh.harvard.edu/) and AFNI (https://afni.nimh.nih.gov/) software tools. MRI scans and segmentations were manually checked for quality and any segmentation errors hand edited. A centroinferior gray matter cerebellar region that excluded vermis to limit specific binding as well as lateral/superior parasinus regions was used to define a reference region for use in modeling for both ligands.

Using FLIRT software (http://fsl.fmrib.ox.ac.uk/fsl/), each dynamically binned PET frame was rigid-body aligned to a central reference frame in the series to further address interframe head motion. Because of the shorter frame duration and limited anatomical definition in the first three PET frames, these frames were yoked to the fourth frame during this alignment step. Using SPM software (https://www.fil.ion.ucl.ac.uk/spm/software/), anatomical MRI volumes and segmentation maps were coregistered to the series mean PET volume. This allowed delineation of time-activity curves for average activity in the cerebellar reference region, which served as an input function to subsequent modeling and were visually inspected to ensure adequate quality for all subjects. ANTS software (http://stnava.github.io/ANTs/) was employed to conduct spatial warping of MRI volumes (and coregistered PET data) to a standard, MNI space template, and smoothing with a 10 mm Gaussian kernel was achieved with SPM software to improve signal to noise ratios.

For primary analyses of [^18^F]-FDOPA data, the tracer specific uptake rate constant, K_i_, a measure of presynaptic dopamine synthesis capacity, was estimated voxelwise across the striatum with PMOD software (https://www.pmod.com/web/) using the non-invasive graphical linearization approach, which assumes an irreversible component (in this case, tracer engagement with DOPA decarboxylase and [^18^F]-fluorodopamine accumulation) over the course of the scan [21]. The whole striatum was delineated for primary analyses as well as segmented into three bilateral canonical functional subregions (associative, sensorimotor, ventral) as previously described [22], and average K_i_ was calculated for each subregion in addition to the whole striatum.

Following evidence suggesting thalamic D_2/3_ dopamine receptor availability is lower in schizophrenia [15], for [^18^F]-fallypride data, the binding potential estimate, BP_ND_, a measure of D_2/3_ dopamine receptor availability, was calculated voxelwise across the thalamus as well as the striatum using the simplified reference tissue model as implemented in PMOD [23, 24]. The whole thalamus was delineated for initial analyses and subsequently segmented into seven bilateral subregions reflecting distinct nuclei groups [25]: anterior; ventral anterior group; medio-dorsal group; ventral latero-ventral group; ventral latero-dorsal group; pulvinar; and a cluster enclosing the central lateral, the lateral posterior and the medial pulvinar (CLLPMP) [25], with mean BP_ND_ calculated for whole thalamus and its subregions. Additionally, average [^18^F]-fallypride BP_ND_ for whole striatum and striatal subregions as defined above were also calculated.

### Statistical analyses

Associations between striatal K_i_ and polygenic predictor variables were tested using linear modeling in R software, with nuisance covariates for age, sex, and population stratification. Separate analyses were performed for non-TRS and TRS polygenic risk scores with an unadjusted statistical threshold of *p* < 0.05. Additional post-hoc analyses were conducted for TRS polygenic risk that also included non-TRS polygenic risk score in the model in order to confirm TRS results as independent of non-TRS effects. Because of the directional nature of hypotheses (i.e., greater presynaptic dopamine synthesis capacity (K_i_) expected with greater non-TRS risk and less TRS risk), one-tailed p-values are provided for each test.

Post-hoc voxelwise general linear model analyses of K_i_ values to more finely localize results within the striatum were conducted with SPM software (https://www.fil.ion.ucl.ac.uk/spm/software/) using a one-tailed, voxel-wise threshold of *p* < 0.005, uncorrected. Models included nuisance covariates for age, sex, and, for genomic analyses, population stratification. Multiple comparisons corrected, threshold-free cluster enhancement (TFCE) statistics were calculated for these models with the TFCE toolbox for SPM (https://neuro-jena.github.io/software.html/; 10000 permutations, Smith permutation method, E = 0.5/H = 2 weighting).

Secondary analyses of [^18^F]-fallypride data proceeded similarly, with BP_ND_ for the thalamus in addition to striatum as outcome variables. Because published data on TRS do not strongly support directional hypotheses for this ligand two-tailed tests were applied to assess the hypothesis that genetic risk may be associated with D_2/3_ dopamine receptor availability. Following hypotheses-guided regional analyses, exploratory whole-brain voxelwise analyses of [^18^F]-fallypride data were additionally conducted with a statistical threshold of p_TFCE-FWE_ < 0.05.

## Results

### Demographics

#### [^18^F]-FDOPA cohort

In the [^18^F]-FDOPA cohort (N = 187, mean age 36 ± 11 years, 98 women [52%], all of European descent), both non-TRS and TRS polygenic scores were independent of age and sex (non-TRS: age: Pearson’s r = 0.02, *p* = 0.74, sex: t(185) = 0.07, *p* = 0.95; TRS: age: Pearson’s r = −0.05, *p* = 0.50, sex: t(185) = 0.30, *p* = 0.77). Tracer dose was not associated with polygenic scores (non-TRS: τ_b_ = 0.08, *p* = 0.13; TRS: τ_b_ = −0.04, *p* = 0.37).

#### [^18^F]-fallypride cohort

In the [^18^F]-fallypride cohort (N = 104, mean age 38 ± 11 years, 47 women [45%], all of European descent), both non-TRS and TRS polygenic scores were independent of age (non-TRS: Pearson’s r = 0.06, *p* = 0.52; TRS: Pearson’s r = 0.11, *p* = 0.25). Non-TRS polygenic scores were independent of sex (t(102) = 0.48, *p* = 0.64) but TRS polygenic scores were not (t(102) = 2.17, *p* = 0.033). Tracer dose was not associated with polygenic scores (non-TRS: τ_b_ = 0.01, *p* = 0.92; TRS: τ_b_ = −0.07, *p* = 0.31).

### Neuroimaging data

#### [^18^F]-FDOPA cohort

**Fig. 1:**
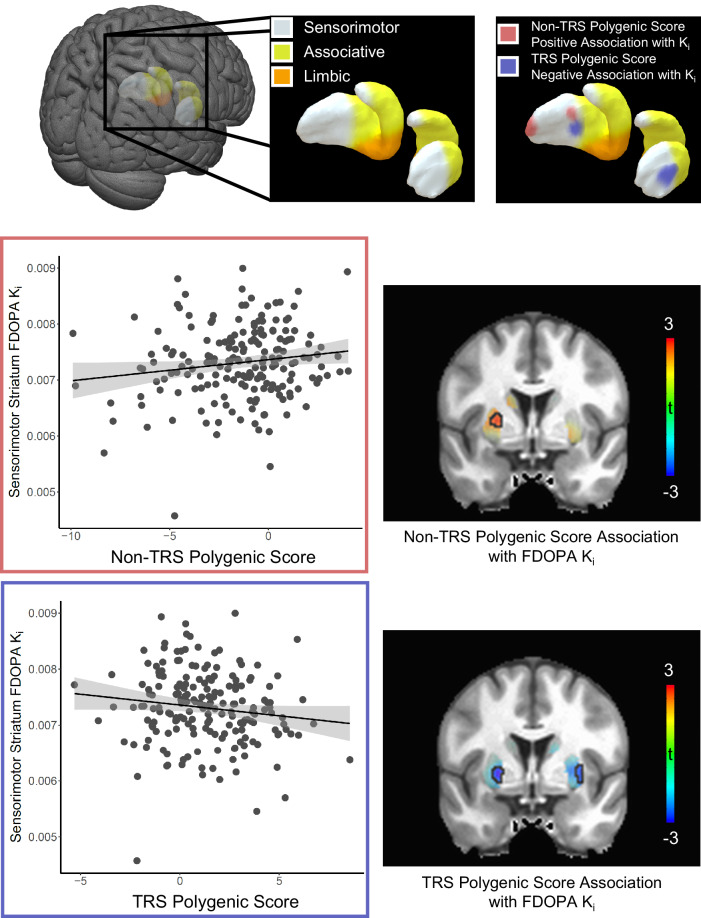
[^18^F]-FDOPA specific uptake (K_i_) results. Top row: Depiction of striatal subregional anatomy (narrowest aspects of caudate tail not visualized), viewed from a right posterosuperior angle. Posterior dorsal sensorimotor striatum shown in gray, anterior dorsal associative striatum shown in yellow, ventral limbic striatum shown in orange. Rightmost images show regions of genetic associations with presynaptic dopamine synthesis capacity ([^18^F]-FDOPA K_i_; *p* < 0.005, uncorrected) overlaid on the striatal surface. Red areas show regions of positive association with non-TRS polygenic risk scores, whereas blue areas show regions of negative association with TRS polygenic risk scores. Lower left: Plots represent whole sensorimotor striatal average [^18^F]-FDOPA K_i_ as a function of non-TRS (red outline) and TRS (blue outline) polygenic risk scores. Linear fits and respective shaded 95% confidence intervals are shown. Lower right: Parametric statistical maps of non-TRS (top) and TRS (bottom) genetic association with presynaptic dopamine synthesis capacity are shown overlaid on a grayscale T1-weighted anatomical MNI space MRI template in the coronal plane (y = 1.5 mm). Colors represent t-values, and results meeting a voxelwise threshold of *p* < 0.005 are outlined in black.

Greater non-TRS polygenic risk was associated with greater [^18^F]-FDOPA specific uptake (K_i_) in the whole striatum (t(180) = 1.86, one-tailed *p* = 0.036). Residuals showed no significant deviation from normality. Subregional analysis revealed this effect to be most evident in the sensorimotor striatum (t(180) = 2.10, one-tailed *p* = 0.018), with associative striatum (t(180) = 1.56, one-tailed *p* = 0.060) and limbic striatum (t(180) = 1.22, one-tailed *p* = 0.11) not demonstrating statistically significant relationships. Post-hoc voxelwise analyses localized results most strongly to left dorsal putamen clusters in both sensorimotor and associative functional subregions of the striatum (Fig. 1).

In marked contrast, greater TRS polygenic risk was associated with less [^18^F]-FDOPA specific uptake (K_i_) in the whole striatum (t(180) = −2.30, one-tailed *p* = 0.011). Residuals showed no significant deviation from normality. This effect was retained even when controlling for non-TRS polygenic risk (t(179) = −2.10, one-tailed *p* = 0.019). Subregional analysis revealed a negative relationship in associative striatum (t(180) = −2.092, one-tailed *p* = 0.019), which was still present when controlling for non-TRS polygenic risk (t(179) = −1.92, one-tailed *p* = 0.028). There was also a negative relationship in the sensorimotor striatum (t(180) = −2.023, one-tailed *p* = 0.022), which was still present when controlling for non-TRS polygenic risk (t(179) = −1.79, one-tailed *p* = 0.038), as well as a negative relationship in the limbic striatum (t(180) = −2.33, one-tailed *p* = 0.011), which was also retained when controlling for non-TRS polygenic risk (t(179) = −2.19, one-tailed *p* = 0.015). Post-hoc voxelwise analyses localized results most strongly to bilateral dorsal putamen clusters in the associative and sensorimotor functional subregions of the striatum; a small focus in the left ventral striatum (i.e., limbic functional subregion) was also identified (Fig. 1).

#### [^18^F]-fallypride cohort

Non-TRS polygenic risk did not show relationships with either whole thalamic [^18^F]-fallypride BP_ND_ (t(97) = 1.63, two-tailed *p* = 0.11) or whole striatal [^18^F]-fallypride BP_ND_ (t(97) = 1.08, two-tailed *p* = 0.28). Residuals showed no significant deviation from normality. Subregional analyses did not yield any statistically significant findings, with positive trends in the anterior group of the thalamus (t(97) = 1.98, two-tailed *p* = 0.050) and the ventral latero-dorsal group (t(97) = 1.76, two-tailed *p* = 0.082) not reaching nominal statistical significance (all other regions, *p*’s > 0.10). Post-hoc voxelwise tests were consistent with these observations (Table 1).

**Table 1:** [^18^F]-FDOPA specific uptake (K_i_) and [^18^F]-fallypride binding potential (BP_ND_) voxelwise results.

Measure	Effect	Direction of effect	Hemisphere	Anatomical region	Functional subregion	Cluster volume (mm^3^)	Peak coordinates (x,y,z)	t value	*p* value
*[*^*18*^*F]-FDOPA*									
	*Non-TRS polygenic risk*								
		Positive	Left	Putamen	Sensorimotor	192	−32, −17, 2	3.32	0.00054
		Positive	Left	Putamen	Associative	415	−29, 6, 5	3.02	0.0014*
	*TRS polygenic risk*								
		Negative	Left	Putamen	Associative/ Sensorimotor	230	−23, 3, −2	3.09	0.0012
		Negative	Right	Putamen	Sensorimotor	270	33, −3, 0	2.93	0.0019*
		Negative	Left	Ventral Striatum	Limbic	20	−8, 18, −5	2.77	0.0031
*[*^*18*^*F]-fallypride*									
	*Non-TRS polygenic risk*								
		Positive	Right	Thalamus	Anterior	84	9, −3, 3	3.12	0.0024
		Positive	Left	Thalamus	Ventral Latero-dorsal	44	−18, −21, 12	3.06	0.0028
	*TRS polygenic risk*								
		Negative	Right	Thalamus	Ventral Latero-ventral	1320	18, −22, 3	3.69	0.00036*
		Negative	Left	Thalamus	CLLPMP	743	−16, −26, 6	3.32	0.0013*

Clusters meeting a voxelwise threshold of *p* < 0.005 (one-tailed for [^18^F]-FDOPA, two-tailed for [^18^F]-fallypride) with greater than 5 voxels in parametric analyses controlling for age, sex, and population stratification are tabulated, with peak coordinates given in MNI space (LPI convention) to the nearest millimeter and uncorrected *p* values shown. Corresponding locales meeting family-wise error (FWE) correction for multiple comparisons (*p*_TFCE-FWE_ < 0.05) are indicated with an asterisk.

**Fig. 2:**
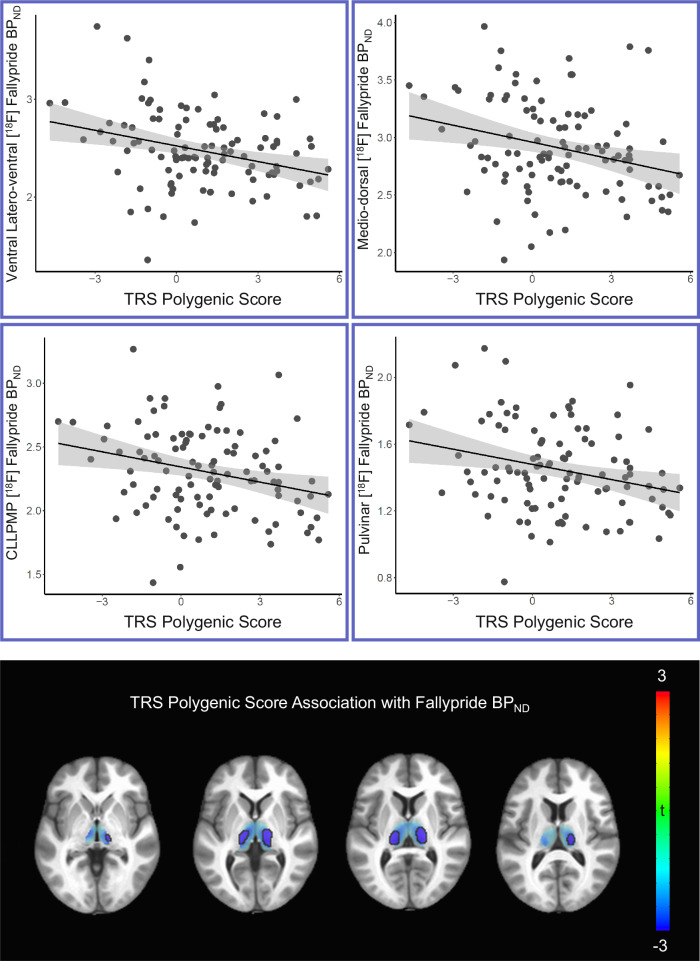
[^18^F]-fallypride binding potential (BP_ND_) results. Top two rows: Plots represent thalamic subregional average [^18^F]-fallypride BP_ND_ as a function of TRS polygenic risk scores. Linear fits and respective shaded 95% confidence intervals are shown. Third row: Parametric statistical maps of TRS genetic association with [^18^F]-fallypride BP_ND_ are shown overlaid on a grayscale T1-weighted anatomical MNI space MRI template in the axial plane (z = -3mm, 1.5 mm, 6 mm, 10.5 mm). Colors represent t-values, and results meeting a voxelwise threshold of *p* < 0.005 are outlined in black.

In contrast, greater TRS polygenic risk was associated with less whole-thalamic [^18^F]-fallypride BP_ND_ (t(97) = −2.48, two-tailed *p* = 0.015), an effect that was retained when including non-TRS polygenic risk in the model (t(96) = −2.28, two-tailed *p* = 0.025). Residuals showed no significant deviation from normality. Subregional analyses identified this effect to be observed in the ventral latero-ventral group (t(97) = −2.79, two-tailed *p* = 0.0064), CLLPMP cluster (t(97) = −2.49, two-tailed *p* = 0.015), medio-dorsal group (t(97) = −2.42, two-tailed *p* = 0.017), and pulvinar (t(97) = −2.39, two-tailed *p* = 0.019), but not the anterior, ventral anterior, or ventral latero-dorsal groups (all *p*’s > 0.2). These results were similar when controlling for non-TRS polygenic risk in the model (i.e., all *p*’s <0.05 for ventral latero-ventral group, CLLPMP cluster, medio-dorsal group, and pulvinar). Post-hoc voxelwise analyses localized results to two large bilateral clusters consistent with subregional results (Fig. 2, Table 1). TRS polygenic risk was not associated with whole striatal [^18^F]-fallypride BP_ND_ (t(97) = −0.39, two-tailed *p* = 0.70). Accordingly, subsequent subregional analyses of the striatum also did not reveal any relationships with TRS polygenic risk (all *p*’s > 0.4). Exploratory whole-brain voxelwise analyses of [^18^F]-fallypride BP_ND_ data did not yield results at a statistical threshold of *p*_TFCE-FWE_ < 0.05.

## Discussion

In this study of a large cohort of healthy participants without psychiatric disorders, we found that greater cumulative genetic risk for unselected schizophrenia (non-TRS) was associated with *greater* striatal presynaptic dopamine synthesis capacity, whereas greater cumulative risk for TRS was associated with *less* striatal presynaptic dopamine synthesis capacity. These predicted opposing relationships in healthy individuals parallel those reported in prior [^18^F]-FDOPA PET work describing mean striatal elevations in non-TRS patient cohorts [3] but reductions in people with TRS [13]. Thus, in aggregate, the common gene variants predisposing to TRS not only have implications for the dopamine system, but they do so in a markedly divergent manner than do those associated with schizophrenia more generally (i.e., non-TRS).

While decades of clinical and experimental findings have established dopaminergic dysfunction in schizophrenia spectrum illness as an incontrovertible element of psychosis psychopathology [4], advances in psychiatric genetics have identified an array of risk variants traversing the genome that implicate diverse neurochemical systems [18]. It is therefore noteworthy that cumulative genetic risk for schizophrenia, which incorporates this broad variation, nonetheless demonstrates association with dopamine synthesis capacity, suggesting that across the non-TRS risk landscape, there is significant mechanistic convergence on dopaminergic pathways. The details of such convergence, both in the subset of contributing genetic markers and in the component molecular pathways at play will be important to define. Notably, our findings do not indicate that etiological routes to schizophrenia spectrum illness are exclusively, or even predominantly, dopaminergic but do provide unique, independent genetic support for a link between dopamine and schizophrenia risk.

The direction of this link in non-TRS, i.e., greater non-TRS genetic risk corresponding to greater presynaptic dopamine synthesis capacity, abides with the observation that on average, individuals with schizophrenia spectrum illness who have been studied with [^18^F]-FDOPA PET show statistically greater striatal K_i_ than controls, especially in associative and sensorimotor subregions [26]. Notably, however, excessive striatal presynaptic dopamine synthesis capacity is not universally observed [11]. In fact, prior work has shown important clinical correlates of this variability in patient samples. For instance, even in the absence of higher presynaptic dopamine synthesis capacity levels, greater severity of negative symptoms – some of the symptoms that are least responsive to treatment – is associated with *less* striatal K_i_ [11]. Furthermore, as noted above, in people with schizophrenia taking clozapine due to inadequate response to other antipsychotic agents, findings of significantly *lower* presynaptic dopamine synthesis capacity [13] relative to healthy controls have raised the possibility that some subgroups of individuals with schizophrenia spectrum illness may have distinct dopaminergic system disruptions, though there has been little experimental evidence to clarify to what factors this divergence is owed.

In the current study, greater loading for genetic markers that were differentially associated with TRS was associated with lower striatal K_i_, a finding that suggests that TRS risk mechanisms include substantive perturbation of dopaminergic systems. While the previously published genetics of TRS risk do implicate a number of diverse neurochemical routes to treatment resistance [14], our findings are contrary to proposals that TRS is an entirely non-dopaminergic entity. Additionally, the direction of the association, in accord with this study’s hypotheses and prior clinical literature [12], provides an important counterpoint to the notion that TRS should necessarily be conceptualized as simply more severe illness along a singular psychosis dimension [27]. Instead, the current results prompt speculation that at least some of the lower striatal K_i_ observed in individuals with histories of clozapine and first-line treatment failures may be attributable to etiological TRS neurobiology rather than medication effects or other epiphenomena.

Evaluation of [^18^F]-fallypride data found an absence of genetic associations in the striatum, suggesting that associations between striatal presynaptic dopamine synthesis capacity and cumulative schizophrenia genetic risk are not clearly accompanied by colocalized genetically-driven biases in striatal D_2/3_ dopamine receptor availability. In schizophrenia, reported differences in striatal D_2/3_ dopamine receptor availability have been relatively weak when compared to findings from the presynaptic dopamine synthesis capacity literature [3]. However, recent metanalytic work has identified a potentially important extrastriatal D_2/3_ receptor phenotype wherein diminished thalamic D_2/3_ receptor availability, measured with high-affinity tracers such as [^18^F]-fallypride, is associated with schizophrenia [15]. Despite dopamine supersensitivity hypotheses that posit compensatory increases in D_2/3_ availability promoting antipsychotic resistance for some individuals [28], D_2/3_ molecular neuroimaging findings in TRS have yet to firmly establish a replicable biomarker for treatment resistance. Nonetheless, the fact that in healthy individuals, whose D_2/3_ receptor availability is unencumbered by antipsychotic treatment histories or other potential illness-associated confounds, thalamic [^18^F]-fallypride binding potential was inversely related to TRS associated genetic variation provides further evidence for a dopaminergic aspect of treatment-resistant neurobiology. Given D_2/3_ targeting of antipsychotic agents, future studies of individuals with schizophrenia will be important to understand whether a genetically-driven shift in thalamic dopamine circuit operations might be related to an individual’s propensity for treatment resistance.

The dopamine system and its modulation due to illness risk, illness itself, and treatment are complex and inadequately understood. It is unclear, for instance, where in the cascade of molecular events from compounding genetic and environmental risk factors to clinical manifestations dopaminergic dysfunction lies. The possibility that dopaminergic pathophysiology may be substantively different in TRS relative to non-TRS deepens the complexity of this knowledge gap. Thus, whether and how TRS risk associated biases in dopamine system functions might modify treatment response remains unknown and merits further study. Nonetheless, the present results suggest that the balance of non-TRS and TRS risk biology may meaningfully contribute to dopaminergic functioning. To the extent that such functioning is also relevant to clinical heterogeneity and treatment resistance in schizophrenia spectrum illness [11, 12], these results provide further impetus to test whether dopaminergic imaging may be valuable in characterizing and stratifying clinical cohorts for treatment trials. Additional work to understand whether dopamine synthesis capacity reductions in TRS risk and TRS are consequential in therapeutic response or simply downstream sequelae of a unique molecular risk signature will be important to drive biomarker development and treatment targeting.

The preponderance of presynaptic dopamine synthesis capacity findings localized to dorsal striatal regions is consistent with notions of the importance of this region in schizophrenia [26]. Though small amounts of signal in limbic striatum suggest the possibility of important ventral involvement as well, more definitive studies using higher resolution tomographs or perhaps complementary ex vivo studies will be needed to better determine relative contributions of subregional circuits to these effects. Given topographically defined distinct connectivity patterns across corticostriatal networks [29], such studies may direct further discovery of mechanisms giving rise to previously observed striatal dopamine synthesis capacity clinical associations.

The D_2/3_ receptor findings were greatest in several regions of the thalamus previously implicated in schizophrenia, including medio-dorsal regions where individuals with schizophrenia show less D_2/3_ receptor availability [15]. Given the dense, neuroanatomically complex nature of the thalamus, additional work with higher resolution imaging techniques to more precisely define the site of TRS relevant thalamic biology will be important. Together with dopamine synthesis capacity results, these findings provide additional support for the relevance of thalamostriatal dopaminergic circuitry in the mechanisms underlying genetically driven treatment resistance in schizophrenia.

This study has several limitations. First, although the study of healthy volunteers permitted examination of TRS and non-TRS genetic risk effects while avoiding many of the confounds present in patient samples, these data cannot speak directly to disturbances observed in individuals with schizophrenia spectrum illness, and further studies in TRS and non-TRS groups will be needed to fully understand the clinical relevance of the present findings. While the European background of participants in this study facilitated the use of polygenic scores derived from genome-wide association studies of mostly European samples, it is essential to extend work of this nature to groups of more diverse ancestry. Because [^18^F]-fallypride has affinity for both D_2_ and D_3_ dopamine receptors, the present work cannot discriminate independent receptor subtype effects. Thus, investigations employing high affinity, subtype-selective ligands or competitive blockade will be useful to further clarify thalamic findings. Additionally, the current PET methods, while well-validated and designed to be both consistent with prior non-TRS and TRS [^18^F]-FDOPA and [^18^F]-fallypride literature, do not include additional assessments for tracer metabolites and arterial input functions, and future studies including these may allow for additional parameter calculations and potentially finer characterization of genetic effects. Finally, unmeasured effects, such as those due to endocrinological, nutritional, or other environmental factors, cannot be ruled out.

In summary, this work provides clear evidence for hypothesized, diametrically opposed associations between striatal dopamine synthesis capacity and both TRS and non-TRS cumulative genetic risk, as well as exploratory D_2/3_ receptor findings in thalamus that may guide future research. These findings align with observations in individuals with schizophrenia spectrum illness and suggest that the molecular underpinnings of treatment resistance include a fundamentally divergent mechanism from that of non-TRS illness risk.

## Data Availability

The data for this study are not publicly available due to consent and regulatory limitations. Some data may be available from the corresponding author on reasonable request. Requests may be subject to review by the NIH Institutional Review Board.
